# Early hypersynchrony in juvenile PINK1^−^/^−^ motor cortex is rescued by antidromic stimulation

**DOI:** 10.3389/fnsys.2014.00095

**Published:** 2014-05-21

**Authors:** Romain Carron, Anton Filipchuk, Romain Nardou, Abhinav Singh, Francois J. Michel, Mark D. Humphries, Constance Hammond

**Affiliations:** ^1^Aix Marseille UniversitéMarseille, France; ^2^Institut National de la Recherche Médicale et de la Santé, INMED, UMR 901Marseille, France; ^3^APHM, Hopital de la Timone, Service de Neurochirurgie Fonctionnelle et StereotaxiqueMarseille, France; ^4^Instituto de Neurociencias, CSIC and Universidad Miguel Hernández, San Juan de AlicanteAlicante, Spain; ^5^B&A Therapeutics, at INMEDMarseille, France; ^6^Faculty of Life Sciences, University of ManchesterManchester, UK

**Keywords:** familial Parkinson, deep brain stimulation (DBS), synchronization, cortical network, antidromic activation

## Abstract

In Parkinson’s disease (PD), cortical networks show enhanced synchronized activity but whether this precedes motor signs is unknown. We investigated this question in PINK1^−^/^−^ mice, a genetic rodent model of the PARK6 variant of familial PD which shows impaired spontaneous locomotion at 16 months. We used two-photon calcium imaging and whole-cell patch clamp in slices from juvenile (P14–P21) wild-type or PINK1^−^/^−^ mice. We designed a horizontal tilted cortico-subthalamic slice where the only connection between cortex and subthalamic nucleus (STN) is the hyperdirect cortico-subthalamic pathway. We report excessive correlation and synchronization in PINK1^−^/^−^ M1 cortical networks 15 months before motor impairment. The percentage of correlated pairs of neurons and their strength of correlation were higher in the PINK1^−^/^−^ M1 than in the wild type network and the synchronized network events involved a higher percentage of neurons. Both features were independent of thalamo-cortical pathways, insensitive to chronic levodopa treatment of pups, but totally reversed by antidromic invasion of M1 pyramidal neurons by axonal spikes evoked by high frequency stimulation (HFS) of the STN. Our study describes an early excess of synchronization in the PINK1^−^/^−^ cortex and suggests a potential role of antidromic activation of cortical interneurons in network desynchronization. Such backward effect on interneurons activity may be of importance for HFS-induced network desynchronization.

## Introduction

Exaggerated resting-state synchronization of oscillatory activities in the sensorimotor cortex notably at β frequency (13–30 Hz) is positively correlated with Parkinson’s disease (PD) severity and attenuated together with motor symptoms by deep brain stimulation (DBS) of the subthalamic nucleus (STN-HFS; Brown and Marsden, 2001; Silberstein et al., 2005; Eusebio et al., 2012; Whitmer et al., 2012). These data were reproduced in the anesthetized or freely moving rodent or primate models of PD (Goldberg et al., 2002; Sharott et al., 2005) validating the links between hypersynchronization and motor symptoms. Yet it is still not known whether cortical hypersynchronization is first observed when motor signs are already present or precedes them.

We used the PINK1^−^/^−^ mice model of PD to determine whether juvenile (P14–P21) motor cortex networks are hypersynchronized and typify the effect of parkinsonian treatments on this signature. The PINK1^−^/^−^ mice is a model of the autosomal recessive PARK6-linked Parkinsonism, an early-onset variant of familial PD caused by loss-of-function mutations in the mitochondrial protein PINK1 (Bentivoglio et al., 2001). PINK1^−^/^−^ mice show electrophysiological signs of dopaminergic dysfunction already at the age of 3–6 months and a reduction of locomotor activity 10 months later (Kitada et al., 2007; Dehorter et al., 2012). This model is therefore highly relevant for investigating the time course between hypersynchronization and motor signs. We focused our study on juvenile mice because the shift from immature to mature cortical activities occurs during the second post natal week (Allene et al., 2008; Dehorter et al., 2011) and thus constitutes the earliest possible stage at which dysfunction of M1 activities can occur. We used two-photon calcium imaging techniques to record the activity of large neuronal populations simultaneously in M1 and a horizontal tilted slice where the motor cortex M1 and the STN were connected via the hyperdirect cortico-STN pathway only.

We report excessive correlation and synchronization in PINK1^−^/^−^ M1 cortical networks as early as 15 months before motor impairment. The percentage of correlated pairs of neurons and their strength of correlation were higher in the PINK1^−^/^−^ M1 than in the wild type network and synchronized network events involved a higher percentage of neurons. Both features were independent of thalamo-cortical pathways, insensitive to chronic levodopa treatment of pups, but totally reversed by high frequency stimulation of the STN (STN-HFS). HFS-evoked cortico-subthalamic spikes propagate antidromically and block orthodromic spikes of M1 pyramidal neurons. They also activate, via axon collaterals, cortical GABAergic interneurons. This last observation points to the potential role of antidromically activated cortical interneurons in network desynchronization.

## Materials and methods

### Slice procedure

All animal experiments were carried out in accordance with the European Communities Council Directive of 24 November 1986 (86/609/EEC). The experimental protocols were carried out in compliance with institutional ethical committee guidelines for animal research, and all efforts were made to minimize the number of animals used and their suffering. We performed experiments in postnatal (P14–P16) wildtype 129 SvEv (Janvier, France) or PINK1 KO 129 SvEv (Gispert et al., 2009) mice of either sex. We performed some of the electrophysiological experiments in (P14–P16) GAD67-green fluorescent protein-knock-in (GAD67-GFP-KI) mice (Tamamaki et al., 2003) to easily identify and record cortical GABAergic interneurons. Mice were killed by decapitation under xylazine (Rompun 2%; used at 0.05%) and ketamine (Imalgene1000; used at 50 g/l; volume injected: 0.2 ml/g) anesthesia. We kept brains in ice-cold oxygenated solution containing (in mM): 110 choline, 2.5 KCl, 1.25 NaH_2_PO_4_, 7 MgCl_2_, 0.5 CaCl_2_, 25 NaHCO_3_, 7 glucose. 400-µm-thick oblique horizontal slices were cut with a vibratome (VT1200 Leica Microsystems Germany) with an angle of 32 ± 2° to obtain the cortico-subthalamic slice. During the recovery period, slices were placed at room temperature (RT) with standard artificial cerebrospinal fluid (ACSF) saturated with 95% O_2_/5% CO_2_ and containing (in mM): 126 NaCl, 3.5 KCl, 1.2 NaH_2_PO_4_, 1.3 MgCl_2_, 2 CaCl_2_, 25 NaHCO_3_, 11 glucose.

### 1,1′-Dioctadecyl-3,3,3′,3′-tetra-methylindocarbocyamine perchlorate (DiI) experiments

**Figure 1:**
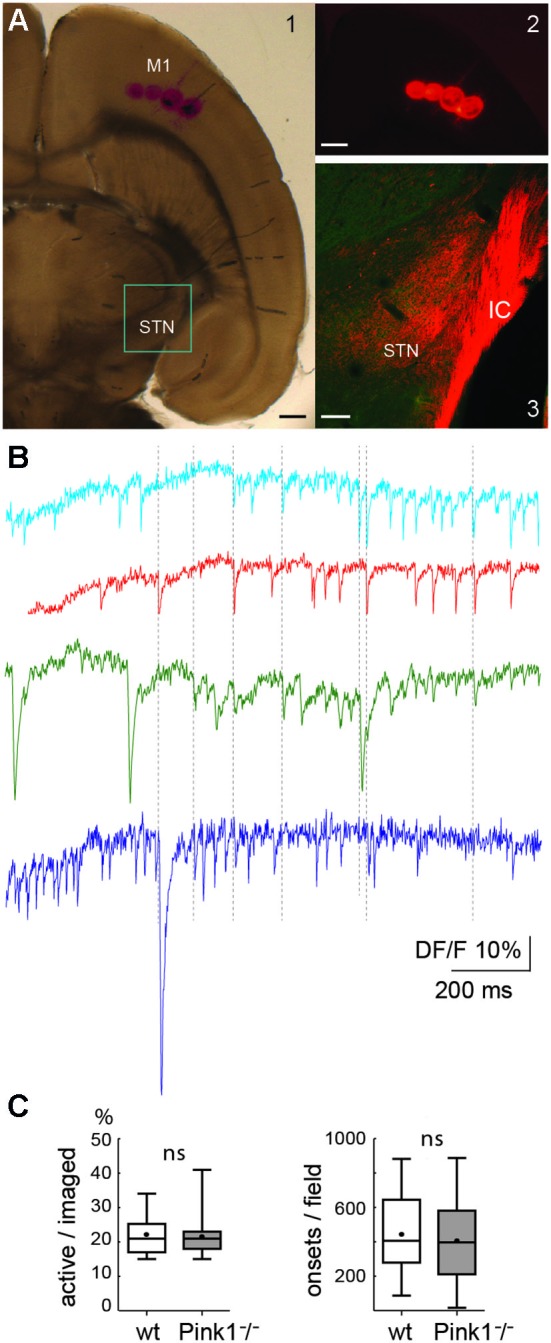
**The cortico-subthalamic slice. (A)** Four DiI injections in M1 cortex in the slice (**A1**, transmission light photo and **A2**, fluorescent image, both just after the injection) and DiI-labeled cortical axons observed 15 days later in the internal capsule (IC) and STN (**A3**, confocal image superposing DiI labeling (red) and Pitx2 labeling (green)). Scale bars A1 and A2: 500 µm; A3: 150 µm. **(B)** Calcium dynamics from a sample of four neurons. Vertical dotted lines indicate some of the synchronous calcium transients. Total duration = 137 s. **(C)** Percent of active cells from all imaged cells (left) and number of onsets of calcium transients from these active cells (right) in wild-type (wt) (*n* = 30) and PINK1^−^/^−^ (*n* = 39) M1 imaged fields. In this and subsequent figures, unless otherwise stated, the bottom and the top of the boxes are the first and third quartiles, the band inside is the median and the point is the mean, the ends of the whiskers are the minimum and the maximum of all the data.

Mice (P14-P16) were anesthetized as above and intracardially perfused with 4% formaldehyde. We postfixed brains by immersion for 2–4 weeks in the fixative solution containing 4% paraformaldehyde and performed 400 µm horizontal sections with an angle of 32 ± 2°. We prepared slices for axon tracing experiments exactly as those used for imaging. We injected small amounts of 1,1′-Dioctadecyl-3,3,3′,3′-tetra-methylindocarbocyamine perchlorate (DiI) (Interchim, France) crystals diluted in ethanol in the M1 cortical region (Figures 1A1–2). Slices were incubated in the fixative solution at 37°C for 2–3 weeks, counterstained with *Pitx2* antibody (*Pitx2* is a homeodomain transcription factor expressed by STN neurons) (Capra Science, Sweden) (Martin et al., 2004), coverslipped and examined with a confocal microscope (Olympus Fluoview-500). For *Pitx2* immunocytochemistry we rinsed slices in PBS and incubated them for 1 h at RT in PBS/Triton 0.3%/goat normal serum 5%. We then incubated them overnight at 4°C with PBS/Triton 0.3%/goat normal serum 5% and pitx2 antibody (1:100) followed by Alexa 488 (1:200, Molecular Probes) in PBS triton for 1 h.

### Two photon calcium imaging

We incubated cortico-subthalamic slices for 30 min in 2.5 ml of oxygenated ACSF (35–37°C) with 25 µl fura 2AM (1 mM, in DMSO + 0.8% pluronic acid; Molecular Probes). We performed imaging with a multibeam two-photon laser scanning system (Trimscope-LaVision Biotec) coupled to an Olympus microscope with a high numerical aperture objective (20X, NA 0.95, Olympus). We acquired images of the scan field (444 µm × 336 µm) via a CCD camera (4 × 4 binning; La Vision Imager 3QE) with a time resolution of 137 ms (non-ratiometric 1000 images, laser at 780 nm) as previously described (Crépel et al., 2007; Dehorter et al., 2011). We previously estimated that around 10% of fura2-loaded cells were astrocytes (Dehorter et al., 2011). These fura2-loaded astrocytes are silent and are thus erroneously counted as silent neurons. This introduces a negligible error in the calculation of the percentage of active neurons.

### Patch clamp recordings and neurobiotin labeling of recorded cells

Cells were visualized with infrared-differential interference contrast optics (Axioskop2; Zeiss). We performed patch-clamp recordings in the whole cell configuration at 35–37°C using the Digidata 1344A interface, the Multiclamp 700A amplifier and PClamp8 software (Axon Instruments, Foster City, CA, USA). For current-clamp recordings, patch electrodes (6–10 MΩ) contained (in mM): 120 K-gluconate, 20 KCl, 10 HEPES, 2 MgATP and 0.5 NaGTP, pH 7.2–7.4 (285–295 mOsm). Biocytin (Sigma-Aldrich; 5 mg/ml) was added in the pipette solution and osmolarity corrected when necessary. To reveal the biocytin injected during whole-cell recordings of pyramidal neurons or interneurons, the recorded sections were fixed in paraformaldehyde (Antigenfix, 4%) for 48 h. They were rinsed in PBS and incubated for 1 h at RT in PBS/Triton 0.3%/goat normal serum 5%. They were incubated overnight at 4°C with PBS/Triton 0.3%/goat normal serum 5% and streptavidin Cy3 (1:1000). They were rinsed in PBS 10 min 5 times, mounted in Eukitt (Electron Microscopy Sciences), and coverslipped. Dendritic fields were reconstructed using the Neurolucida system (MicroBrightField Inc., Colchester, VT, USA).

### Stimulation

Antidromic spikes were evoked in M1 cortical neurons using a bipolar stimulating electrode (1 MΩ impedance, reference TS33A10KT, WPI FL-USA) placed at the antero-lateral border of the STN, close to the internal capsule fiber tract. Rectangular pulses of fixed duration (100 µs) and 20–50 µA amplitude were delivered between the two poles of the electrode at a frequency of 0.1–100 Hz STN (Grass stimulator). 100 Hz stimulations were applied over periods of several minutes.

### Chronic levodopa Treatment

We treated PINK1^−^/^−^ pups over a 14 day period (from P1 to P14) with a daily injection of benserazide hydrochloride (DLserine 2-(2,3,4-trihydroxybenzyl)hydrazide hydrochloride, 10 mg/kg, i.p.), a DOPA decarboxylase inhibitor that does not cross the blood–brain barrier, 20 min delayed levodopa injection (10 mg/kg, i.p.).

### Drugs

Drugs were prepared as concentrated stock solutions and diluted in ACSF for bath application immediately prior to use. Gabazine, a selective GABA_A_ receptor antagonist; (±)-2-amino-5-phosphonopentanoic acid (AP5, a selective NMDA receptor antagonist), 6-cyano-7nitroquinoxaline 2, 3-dione (CNQX, an AMPA-KA receptors antagonist), DL-Serine 2-(2,3,4-trihydroxybenzyl)hydrazide hydrochloride (benserazide), an inhibitor of peripheral dopa decarboxylase and α-methyl-L-tyrosine (levodopa) were all purchased from Sigma Aldrich (France).

### Data analysis

Fluorescent traces were analyzed with custom developed routines in Matlab and calcium events identified using asymmetric least squares baseline and Schmitt trigger threshold. We took into account only fields where more than 15% of fura2-loaded cells were active to ensure sufficient numbers of active cells to detect network-wide synchronization if present. We used 5% of baseline noise as high threshold and 2% as low threshold. Onset and offset identification allowed the generation of raster plots and the estimation of the extent of cortical network correlation. We applied two main tests: pairwise and group tests. Throughout we define “co-active” as meaning those onset transients occurring simultaneously within ± 1 frame time window (411 ms).

*Pairwise correlation test* detected repeated co-activation between two cells (cell pairs continuously correlated). We estimated the degree of similarity of their spiking patterns using normalized Hamming similarity (Humphries, 2011). Each calcium transient train was divided into discrete bins corresponding to the time frame (137 ms) of recording. In each bin the number 1 was given to the presence of any event onset, and the number 0 for their absence. The resulting binary vector thus recorded the pattern of activity and inactivity of the neuron. For each pair of binary vectors the normalized Hamming similarity was the proportion of bins that differed between the two vectors, given the ± 1 frame time window. The parameter approached 1 as the vectors became equal. In the graphic representation the lines connecting correlated cells have colors from red (high similarity of patterns, normalized Hamming similarity close to 1) to dark blue (low similarity of patterns, Hamming similarity close to 0 but never equal to 0, otherwise these cells could not be considered as correlated; see Figure 1A). To estimate the likelihood that the co-activations might be explained by chance we created random datasets for each active cell using inter-event interval reshuffling (Mao et al., 2001; Feldt et al., 2013). We generated 1000 random data sets for each recording session and computed the pairwise Hamming similarity for all pairs in those data-sets, giving a distribution of 1000 randomized similarity scores for each pair of recorded neurons. The threshold for a significantly correlated pair of recorded neurons was set to the 95th percentile of that distribution.

Separately we characterized the existence of *neural ensembles*, i.e., groups of neurons with consistently correlated activity. To do so, each recording was analyzed using the method in Humphries (2011), which we briefly describe here. A similarity matrix was constructed using the computed Hamming similarity between each pair of retained neurons. Note that we do not set a threshold for correlation here, as we wish to retain all information about the correlation structure of the network. This matrix was then partitioned into groups using the community detection algorithm detailed in Humphries (2011), which self-determines the number and size of groups within the matrix that maximizes the benefit function *Q*_data_ = [similarity within groups] − [expected similarity within groups]. The resulting partition thus corresponded to groups of neurons—ensembles—that were more similar in activity patterns than was expected given the total similarity of each neuron’s activity to the whole data-set. We then ran controls for checking if each clustering was significant compared to randomized data. For each recording we generated 20 randomized versions by shuffling each neuron’s inter-event intervals. We clustered each randomized version using the algorithm above, giving us 20 values of *Q*_control_. These allow us to estimate whether or not the actual recording’s *Q*_data_ value exceeded the 95% confidence level: thus a recording was deemed significant at this level if *Q*_data_ > max (*Q*_control_).

The *group event test* identified incidences where a significant fraction of the network’s cells were active together. The minimal size of these groups for each recording session was defined by the corresponding simulated random sets: for each of the 1000 random datasets we counted the number of co-active cells within each time-window; this defined a distribution of expected counts of co-active cells due to random activity. The threshold for significant group co-activation was set at the 95th percentile of this distribution. Such clusters of correlated events were called network events.

In our analyses, we did not take into account fields showing a clear rhythmic pattern reminiscent of the giant depolarizing potentials (GDP) pattern (9% of the wild-type (wt) and 11% of all the PINK1^−^/^−^ M1 recorded fields). This GDP-like pattern consisted of regularly-spaced network events occurring at a frequency of around 0.1–0.3 Hz in wt and PINK1^−^/^−^ M1 networks and was identical in wt and PINK1^−^/^−^ M1 networks. We removed it because it represents the last step in the sequence of immature network activities previously described in the cortex, hippocampus and striatum (Crépel et al., 2007; Allene et al., 2008; Dehorter et al., 2011).

### Statistics

Average values are presented as means ± SEM and we performed statistical comparisons with Mann–Whitney rank sum test (Prisme^TM^). We set the level of significance as (*) for *P* < 0.05; (**) for *P* < 0.01; (***) for *P* < 0.001.

## Results

### The corticosubthalamic slice and spontaneous calcium transients

The 32° tilted horizontal slices contained portions of the M1 cortical region and STN (Figure 1). We checked the presence and functionality of M1 corticofugal axons to the STN with two methods. We visualized M1 corticofugal axons using the axonal tracer DiI (Figures 1A1, 2), or tested their functionality by stimulating the STN area and recording the responses (orthodromic or antidromic) in M1 pyramidal neurons (layers V/VI) (see Figure 5). For technical reasons, DiI and electrophysiological experiments were conducted in different slices cut exactly the same way. Fifteen days after DiI injection in the M1 region, labeled corticofugal axons were seen running in numerous bundles radially through the whole striatum, and in the internal capsule (*n* = 22/22 slices). In the STN DiI labeling was seen around pitx2-positive somas (Figure 1A3). In PINK1^−^/^−^ slices, stimulation of the rostral pole of the STN in control ACSF did not evoke EPSPs or orthodromic spikes in the recorded M1 pyramidal neurons (*n* = 5) whereas it clearly evoked antidromic spikes. We further assessed the antidromic functionality of the cortico-subthalamic connection by recording antidromic spikes in M1 pyramidal neurons in the continuous presence of blockers of ionotropic glutamate receptors and their effect on the network (see Figure 5A). The above results showed that cortico-subthalamic axons are present and functional in the cortico-subthalamic slice.

**Figure 2:**
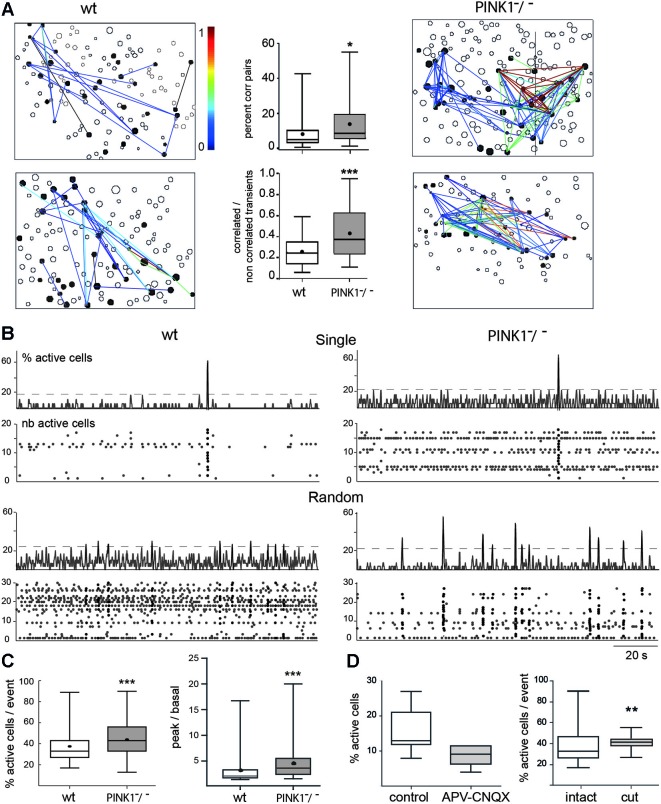
**Spontaneous correlated calcium transients in M1 cortex: correlated cell pairs and network events. (A)** Correlation maps of fura2-loaded M1 cortical cells in four different fields, two from wt (left column) and two from PINK1^−^/^−^ (right column) slices. Each black contour represents a fura2-loaded silent cell and each black-filled contour represents a fura2-loaded active cell. Lines link active cells with a statistically significant correlation (see Section Materials and Methods). Line color is proportional to the value of the correlation (color scale from 0 to 1, right). In wt M1, 20 (top left) or 32 (bottom left) calcium transients are correlated between some of the 30 or 33 active cells, respectively. In contrast in PINK1^−^/^−^ M1, 115 (top right) and 98 (bottom right) calcium transients are correlated between most of the 35 or 34 active cells, respectively. (Middle column) Percentage of correlated cell pairs compared to all combinations of fura2-loaded cells (active and non-active) (top). Ratio of the correlated to the non-correlated calcium transients for each active cell pair (bottom). This indicates the similarity of firing between each active cell pair. **(B)** Representative histograms (top traces, % active cells) and raster plots (bottom traces, number of active cells) of the population dynamics after onset detection in four different M1 networks in slices from P14–P16 wt (left column) and PINK1^−^/^−^ (right column) mice. The proportion of fields with singly or randomly distributed network events was for wt: 10–90%, *n* = 30; and for PINK1^−^/^−^: 20–80%, *n* = 39. Randomly distributed network events had a similar frequency in wt (0.034 ± 0.016 Hz, *n* = 27 fields) as in PINK1^−^/^−^ (0.03 ± 0.02 Hz, *n* = 30 fields) M1 networks (*P* = 0.9). **(C)** Percentage of active cells involved in network events (% active cells/ network event) (left) and ratio of the percentage of active cells inside (peak) and outside (basal) network events in wt (*n* = 30) and PINK1^−^/^−^ (*n* = 39) M1 fields that displayed single or random network events (right). **(D)** Percentage of active cells in wt M1 before (control) and during application of APV and CNQX (left) and percentage of active cells involved in network events before (intact) and after cutting (cut) the slices between cortex and thalamus.

**Figure 3:**
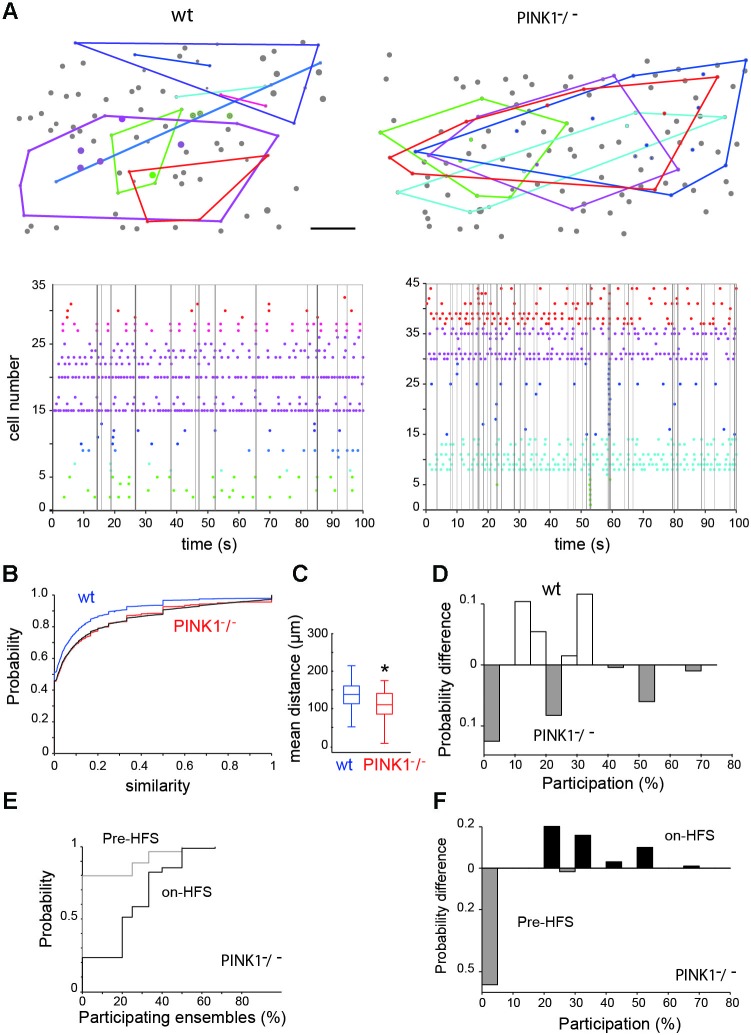
**Spontaneous neural ensembles in M1 wt and PINK1^−^/^−^ cortex. (A top)** Spatial distribution of the ensembles in wt (left) and PINK1^−^/^−^ (right) M1 networks. A polygon has been overlaid to enclose all cells in each ensemble. Scale bar: 50 µm. **(A bottom)** Representative raster plots of population activity containing neural ensembles in the same wt (left) and PINK1^−^/^−^ (right) M1 networks. Calcium transients are color-coded by ensemble membership. Vertical gray lines indicate detected synchronized network events. **(B)** Cumulative distributions of pairwise similarity values across all recordings. The black line gives an example transformed wt distribution. **(C)** Distributions of the mean physical distance between all neurons in an ensemble. Center lines give the median, boxes give the inter-quartile range, and whiskers extend to 1.5 times that range. **(D)** Histogram of the difference between the wt and PINK1^−^/^−^ networks in the probability of ensemble participation in network events. Participation is defined as the percentage of ensembles with more than half their members active during the network event. **(E)** Cumulative distributions of ensemble participation in network events before and during HFS. **(F)** Histogram of the difference between before-HFS and during-HFS in the probability of participation, showing that HFS increased ensemble participation in network events.

**Figure 4:**
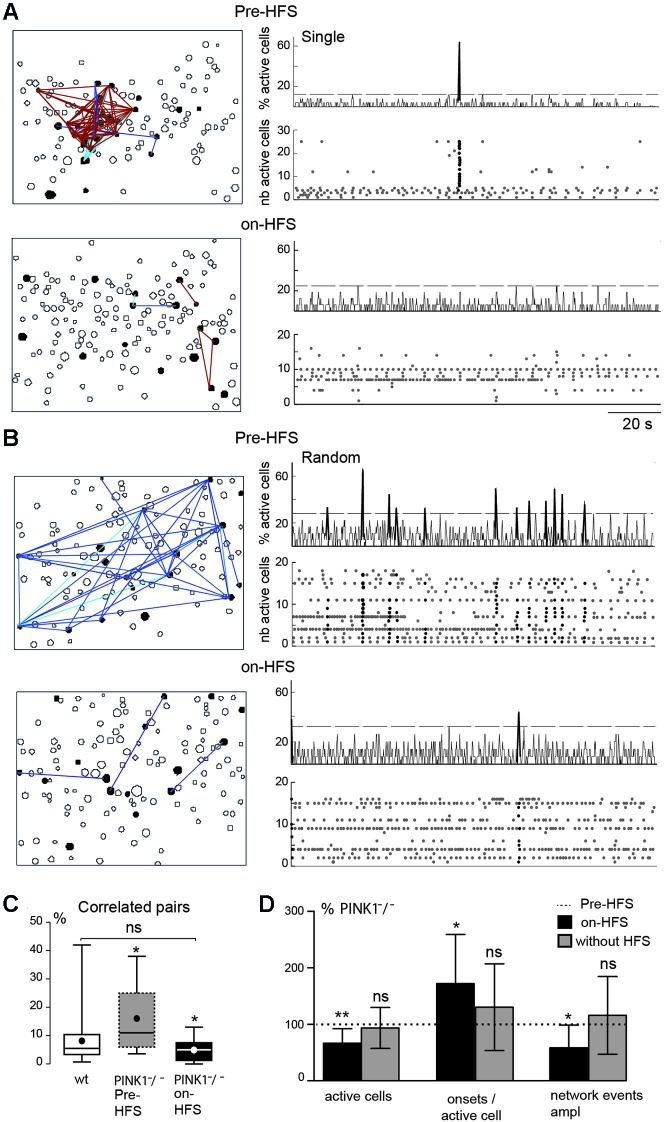
**M1 correlated cell pairs and network events during STN-HFS. (A, B)** Example of two PINK1^−^/^−^ M1 fields. Left are correlation maps of the fura2-loaded M1 cortical cells before (Pre-HFS) and during (on-HFS) STN-HFS; right are the corresponding histograms (top traces) and raster plots (bottom traces) of the same population dynamics after onset detection before and during STN-HFS (applied for at least 3 min). They display before HFS either a single network event (**A**, Pre-HFS) or randomly distributed network events (**B**, Pre-HFS) (total recording 137 s). **(C)** Percentage of correlated cell pairs compared to all combinations of fura2-loaded cells (active and non-active) in wt (*n* = 35) or PINK1^−^/^−^ M1 (*n* = 9) fields before and during HFS as indicated. **(D)** From left to right, percentage of active cells, onsets per active cells, network event amplitude in M1 fields after 3 min HFS (black, *n* = 9 PINK1^−^/^−^ M1 fields) or after 3 min without HFS (gray, *n* = 27 PINK1^−^/^−^ M1 fields), compared to the same items before HFS (normalized at 100%, dotted line).

**Figure 5:**
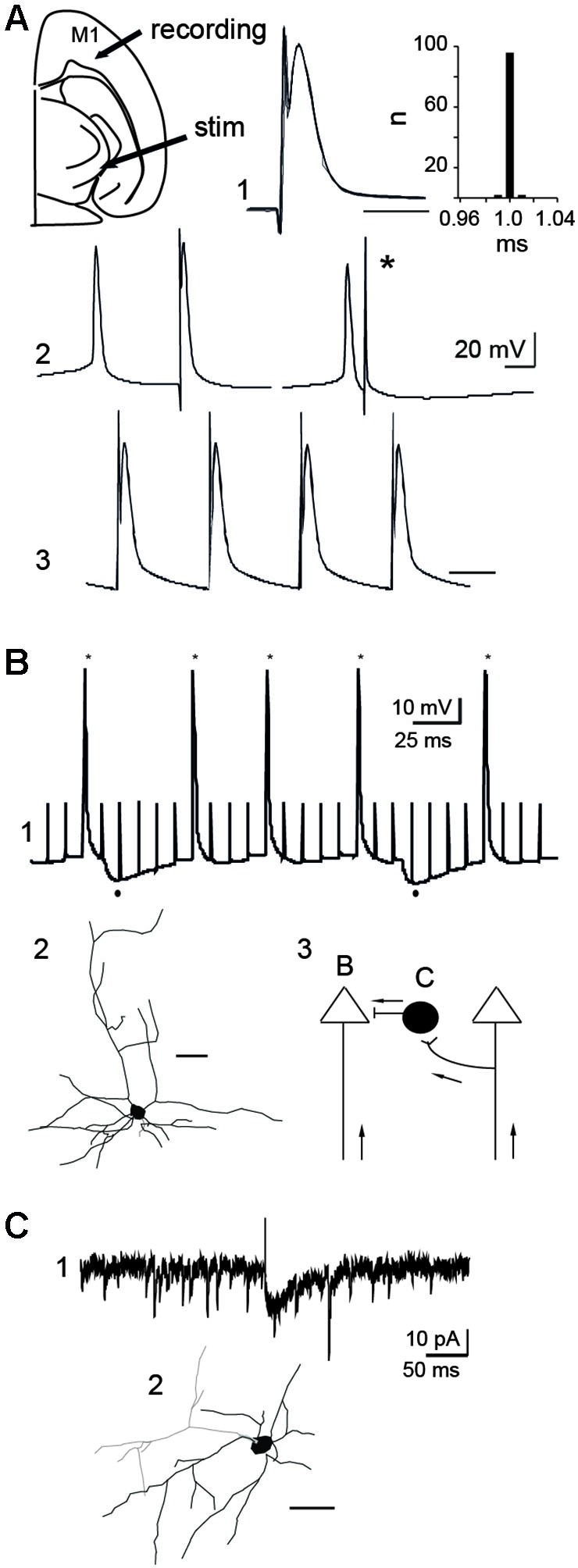
**Mechanisms of action of STN-HFS. (A)** Antidromic activation of a PINK1^−^/^−^ M1 pyramidal neuron in response to rostral STN stimulation in the continuous presence of APV (40 µM) and CNQX (10 µM). Schematic illustration of the stimulation and recording sites (top left). Five superimposed traces and latency histogram **(A1)**, collision test **(A2)** and 100 Hz stimulation **(A3)**, respectively showing the fixed latency of the antidromic spike, its collision (*) with a spontaneous spike and its ability to follow a short train of HFS (*V*_m_ = −60 mV, scale bars: 2 ms). **(B)** Whole cell recording **(B1)** of a PINK1^−^/^−^ pyramidal neuron in control ACSF (current clamp mode, *V*_m_ = −50 mV). Stars indicate the antidromic spikes and dots below indicate the two hyperpolarizing responses recorded in response to some of the stimuli (100 Hz). **(B2)** Biocytin-filled, whole-cell recorded M1 pyramidal neurons of layers V-VI that responded to antidromic stimulation (scale bar 50 µm). **(B3)** Schematic illustration of two pyramidal neurons and one interneuron to show recording sites in B and C; Arrows indicate direction of propagation of STN-HFS evoked antidromic spikes. **(C)** Whole cell recording of an inward glutamatergic current **(C1)** evoked by the stimulation of the rostral STN (100 µs, 0.1 Hz) in a cortical GAD-GFP interneuron (**C2**, scale bar: 50 µm) (voltage clamp mode, *V*_H_ = −70 mV, in the continuous presence of gabazine 10 µM).

Single neurons (projection neurons and interneurons) of the deep layers (V/VI) of the M1 cortex in slices from aged-matched wt or PINK1^−^/^−^ mice generated spontaneous calcium transients in control ACSF (Figure 1B). The voltage-sensitive sodium channel blocker tetrodotoxin (TTX, 1 µM) or the calcium channels blocker Cd^2+^ (200 µM) blocked all calcium transients (not shown) indicating that they resulted from the opening of voltage-gated calcium channels by sodium action potentials. On average, the ratio of active cells to the number of fura2-loaded cells was similar in wt (22 ± 5%, *n* = 709 out of 3085 neurons in 30 fields) as in PINK1^−^/^−^ (22 ± 6%, *n* = 1034 out of 4763 neurons in 39) M1 networks (*P* = 0.4). Also, the average number of identified onsets of calcium transients per field (1000 images) was similar in wt (444 ± 222 per 137 s, *n* = 709 active neurons in 30 fields) as in PINK1^−^/^−^ (406 ± 231 per 137 s, *n* = 1034 active neurons in 39 fields) M1 active neurons (Figure 1C). To disambiguate our terminology, we henceforth refer to the onsets of calcium transients for individual neurons as “calcium transients”, reserving the term “network events” for synchronized transient onsets at the network-level.

### Pair-wise correlations and spontaneous network events

PINK1^−^/^−^ M1 networks contained a significantly higher percentage of correlated pairs of cells (14 ± 11%, *n* = 4873 neurons in 40 fields) than wt M1 networks (8 ± 8%, *n* = 3541 neurons in 35 fields) (*P* = 0.01). The strength of correlation, represented by the similarity of patterns of calcium transients between pairs of cells (1 means that patterns were totally similar and simultaneous), was also significantly higher in PINK1^−^/^−^ than in wt M1 networks: 0.44 ± 0.24 (*n* = 40 fields) vs. 0.26 ± 0.14 (*n* = 35 fields), respectively (*P* = 0.0005) (Figure 2A). In the majority of M1 slices from wt (87%) and PINK1^−^/^−^ (98%) mice we identified synchronized network events characterized by statistically significant synchronous network-level activity (coactive within a 411 ms time window). Their time distribution had two main patterns: network events at very low frequency (0.007 Hz), which we named single, and irregularly spaced network events synchronized at low frequency (0.03 Hz), which we named random (Figure 2B). Both types of network events involved a significantly higher percentage of active cells in PINK1^−^/^−^ (45 ± 18%, *n* = 140 events in 39 fields) than in wt (38 ± 15%, *n* =123 events in 30 fields) M1 networks (*P* = 0.0004). Also, there was a significantly higher proportion of active cells involved in network events (signal) than in basal activity (noise) in PINK1^−^/^−^ (4.4 ± 3.0) than in wt (3.1 ± 2.6) M1 networks (*P* < 0.0001; Figure 2C). Therefore, the activity is more concentrated in network events in PINK1^−^/^−^ than in age matched wt M1 networks.

Antagonists of ionotropic glutamate receptors (CNQX-APV, 10–40 µM) decreased by 50% the number of active M1 neurons (from 16 ± 6% to 8 ± 3%, *n* = 7, *P* = 0.012) and abolished all network events (Figure 2D). Subsequent application of the GABA_A_ antagonist gabazine (10 µM) totally abolished spontaneous activity. In contrast, cutting the slices between thalamus and M1 networks to interrupt thalamo-cortical pathways slightly decreased the percentage of spontaneously active cells (22 ± 5% in control and 17 ± 5% in cut slices, *P* = 0.03), but did not affect the percentage of correlated pairs (8 ± 8% in control and 6 ± 5% in cut slices, *P* = 0.64) and increased the percentage of cells involved in network events (38 ± 15% in control and 41 ± 6% in cut slices, *P* = 0.008) (*n* = 30 M1 fields and 120 events in control slices and *n* = 8 M1 fields and 29 events in cut slices; Figure 2D). Therefore, pair-wise correlations and network events are driven by local circuit synapses.

### Neural ensembles

The shuffled controls showed that 28% (10/35) of wt and 30% (12/40) of PINK1^−^/^−^ networks were organized into ensembles of consistently co-active neurons (Figure 3A). We found no significant difference between the wt and PINK1^−^/^−^ ensembles for average discreteness (*Q* values), average number of ensembles, or average number of neurons per ensemble (*P* > 0.05). However, the ensembles were more tightly grouped in PINK1^−^/^−^ (107 +/− 3.8 µm, *n* = 40) than in wt (141 +/− 8.4 µm, *n* = 38) (*P* = 0.008) networks (Figure 3C). Thus, despite the absence of detectable difference in their functional structure (in their existence, number, size or discreteness) neural ensembles of wt and PINK1^−^/^−^ M1 networks significantly differed in their physical structure. The smaller spatial extent of the ensembles may reflect an anatomical change in pyramidal cell connectivity in the PINK1^−^/^−^ motor cortex (for example a higher probability of synaptic contact between neighbors).

Given the clear difference in significant pairwise correlation between wt and PINK1^−^/^−^ networks (Figure 2A), this lack of differences in ensemble structure was unexpected. To examine this issue, we pooled all calculated Hamming similarity values (significant or not) across recordings to compare the global correlation statistics between wt and PINK1^−^/^−^ M1 cortex. The resulting distributions were very similar (Figure 3B) but translated from each other, suggesting that hypersynchrony in PINK1^−^/^−^ M1 cortex was due to a uniformly-random increase in correlation compared to the wt circuit. Consistent with this hypothesis, we found that randomly increasing ~10% of wt correlation values by a randomly chosen value between 0.05 and 1 perfectly transformed the wt distribution into the PINK1^−^/^−^distribution of correlations (black line in Figure 3B). We thus concluded that the difference of pair-wise correlation between wt and PINK1^−^/^−^ M1 cortex is not related to a difference of ensemble structure but results from a uniformly-random increase in correlation.

To understand the relationship between the neural ensembles and network events, we then examined the participation of ensembles within those network events. Following Feldt et al. (2013), we counted an ensemble as participating if at least half its members were active during the network event. We found that ensemble participation was very weak in both wt (11 +/− 0.5%, *n* = 404 events) and PINK1^−^/^−^ (11 +/− 0.6%, *n* = 497 events) networks; median participation was 0% in both wt and PINK1^−^/^−^ networks (Figure 3D). However, there was a clear difference in the distributions of ensemble participation (*P* = 0.0017, two-sample Kolmogorov-Smirnov test): as Figure 3D shows, PINK1^−^/^−^ networks had a higher probability either of no ensembles participating at all or of the majority of ensembles participating in a network event. Thus, only in PINK1^−^/^−^ networks could network events ever recruit the majority of neural ensembles.

### Chronic levodopa treatment

We next tested whether chronic L-dopa treatment of PINK1^−^/^−^ pups would reverse the excess of synchronization of the PINK1^−^/^−^ M1 network. A deficit of evoked dopaminergic transmission has been identified in the 3 months-old PINK1^−^/^−^ mouse (Kitada et al., 2007; 1058 /id) but when it begins during development is still unknown. All the experiments were performed in PINK1^−^/^−^ slices. L-dopa treatment from P1 to the day of recording (P14–P16) had no significant effect on any of the characteristics of PINK1^−^/^−^ M1 network activity (data not shown). M1 networks from untreated or L-dopa-treated PINK1^−^/^−^ mice contained a similar percentage of correlated pairs of cells (14 ± 11%, *n* = 4873 neurons in 40 fields vs. 17 ± 16%, *n* = 522 neurons in 19 fields) (*P* = 0.5). L-dopa treatment did not significantly change the percentage of PINK1^−^/^−^ M1 fields showing network events (96% treated vs. 98% untreated) and the pattern of these events (27% vs. 20% single, 55% vs. 67% random). The mean frequency of randomly distributed network events was similar in untreated (4.3 ± 2.6 peaks per 137 s or 0.03 ± 0.02 Hz, *n* = 30 fields) as in L-dopa-treated (4.3 ± 3.0 peaks per 137 s or 0.03 ± 0.02 Hz, *n* =12 fields) M1 fields (*P* = 0.9). Single or randomly-distributed network events involved a similar percentage of active cells in PINK1^−^/^−^ M1 fields from untreated (45 ± 18%, *n* = 140 peaks in 39 fields) as from L-dopa-treated (50 ± 21%, *n* = 54 peaks in 18 fields) (*P* = 0.2) PINK1^−^/^−^ mice. Also, the ratio between the percent of active cells involved in basal activity (noise) to that involved in network events (signal) was similar without (0.30 ± 0.15, 140 events in 39 fields) as with (0.28 ± 0.14, *n* = 54 events in18 fields) (*P* = 0.4) L-dopa treatment.

### Cortical desynchronization during STN-HFS

HFS of the antero-lateral pole of the STN (STN-HFS) significantly decreased the percentage of M1 active cells to the number of M1 fura2-loaded cells, from 100% before HFS to 67 ± 26% (*P* < 0.01 paired *t*-test) and significantly increased the average number of onsets of calcium transients per M1 active cell to 170 ± 90% (*n* = 212 active cells in 9 fields, *P* < 0.05 paired *t*-test).

STN-HFS significantly decreased the percentage of correlated cell pairs from 16 ± 12% to 5 ± 4% in PINK1^−^/^−^ M1 fields (*n* = 9 fields with 212 active cells, *P* = 0.03, paired *t*-test), a value not significantly different from the percentage of correlated cell pairs in wt fields (8 ± 8%, *n* = 35, *P* = 0.23 unpaired *t*-test; Figures 4A–C). After the same time of recording (at least 3 min) but without HFS (data not shown), the percent of correlated M1 active cells in non-stimulated PINK1^−^/^−^ slices non-significantly increased (to 185 ± 310%, *n* = 732 active cells out of 3386 imaged cells in 27 fields, *P* = 0.72 paired *t*-test). STN-HFS also significantly decreased by 25% the amplitude of unique or randomly distributed network events in PINK1^−^/^−^ M1 fields (152 active cells out of 768 imaged cells in 7 fields, *P* = 0.03 paired *t*-test). Low frequency (0.1–10 Hz) STN-HFS failed to produce these effects (not shown) that were also not due to the recording duration (Figure 4D). Finally, STN-HFS had no significant effect on the percentage of correlated cell pairs in wt M1 fields (9 ± 6% pre HFS vs. 10 ± 7% during STN-HFS, *n* = 5 fields with 105 active cells, paired *t*-test, *P* = 0.5). Collectively, these observations suggest that STN-HFS produced a de-correlation of PINK1^−^/^−^ M1 networks.

Neural ensembles were present both before (2 out of 9 recordings) and during (4/9 recordings) STN-HFS. STN-HFS did not significantly change the proportion of neurons comprising an ensemble (29 ± 5% of significant ensembles before HFS, *n* = 7 and 29 ± 5%, *n* = 14 during-HFS, *P* = 0.94), the discreteness of the ensembles or the physical size of ensembles. Interestingly, HFS significantly increased the participation of ensembles in network events from 6.5 ± 0.8% per network event before HFS to 24 ± 1% per network event during HFS (*P* ~ 8 * 10^−14^, two-sample Kolmogorov-Smirnov test; see Figures 3E, F).

These changes were due to HFS since after the same recording duration (at least 3 min) without HFS, the percent of M1 active cells non-significantly decreased compared to control (to 94 ± 36%), the number of onsets per active cell non-significantly increased (to 130 ± 78%) (*n* = 732 active cells out of 3386 imaged cells in 27 fields) and the amplitude of network events did not significantly increase with time (to 116% ± 69% of the initial amplitude, *n* = 18 fields, *P* = 0.73 paired *t*-test; Figure 4D).

To study the mechanisms of action, we recorded in whole-cell configuration PINK1^−^/^−^ M1 pyramidal neurons. 100 Hz STN-HFS evoked antidromic spikes that we studied in the continuous presence of CNQX-APV (10–40 µM, *n* = 8/12 neurons). These spikes were antidromic as they were not preceded by EPSPs, had a fixed latency, collided with spontaneous spikes and followed a short train of 100 Hz stimuli (Figure 5A). Therefore, stimulation of the rostral STN at 1–100 Hz evoked antidromic spikes that propagated along cortico-subthalamic axons. When we performed the same experiment in the absence of APV-CNQX we still recorded antidromic spikes but failed to record orthodromic EPSPs in pyramidal neurons in response to STN stimulation (*n* = 0/15). To get rid of the antidromic spike that could occlude short latency EPSPs, we hyperpolarized the recorded pyramidal neuron to *V*_m_ = −80 mV. Even in this configuration where other pyramidal cells were still antidromically invaded, orthodromic excitatory responses were absent. This suggests that functional subthalamo-cortical synapses (Degos et al., 2008) are rare or absent and that recurrent collaterals between pyramidal neurons were not activated by antidromic stimulation.

We next determined whether HFS-evoked antidromic spikes also activate GABAergic interneurons via axonal collaterals of pyramidal cells. We stimulated the STN (100 µs, 100 Hz) and recorded GABA_A_-mediated IPSPs in around 10% of pyramidal neurons (*n* = 1/11, *V*_m_ = −50 mV; Figure 5B), suggesting that a small fraction of GABAergic interneurons was activated by antidromic spikes. This was confirmed in slices from aged-matched wt mice expressing GFP in GAD neurons. The stimulation of the rostral STN (100 µs, 0.1 Hz) in the continuous presence of gabazine (10 µM) to block GABA_A_ receptors, evoked glutamatergic EPSCs from around 10% of GABAergic interneurons (voltage clamp mode, *V*_H_ = −70 mV, *n* = 1/12; Figure 5C). Therefore, HFS-evoked antidromic spikes can also engage GABAergic signals in the effects of STN-HFS.

## Discussion

We show here that PINK1^−^/^−^ cortico-cortical networks are engaged in hypersynchrony at juvenile stage, well before motor symptoms. This early signature is dopamine-insensitive but STN HFS-sensitive. The mechanism of HFS-induced desynchronization includes invasion of the cortical network (pyramidal cells and interneurons) by HFS-generated antidromic spikes. Since, in our preparation, the only connections between M1 and STN originate from layer V/VI to the STN, it appears that the rescue of cortical hypersynchronization results from distal actions of HFS via antidromic axonal spikes. This study provides a first insight into early cortical hypersynchronization of a rodent model of a familial form of PD. Further experimental investigations will be needed to understand how it evolves in the course of the disease, once dysfunction of dopaminergic transmission has begun and dopamine-sensitive synchronizations develop.

The synchronous network-level activity recorded here is reminiscent of cortical up states of the wt mouse primary visual cortex (Mao et al., 2001; Cossart et al., 2003) and of the so-called avalanches, the spatio-temporal clusters of synchronous activity interrupted by periods of low activity described in cultured slices from wt cortex (Beggs and Plenz, 2003; Yang et al., 2012). Pair-wise correlations and network events were synaptically and locally produced since they were blocked by antagonists of ionotropic glutamatergic and GABAergic channels but were still present after the mechanical interruption of the thalamo-cortical pathway. The vast majority of the glutamatergic excitatory synapses originate in the cortex itself, with recurrent excitatory interactions in groups of neurons inducing the slow rhythmic depolarizations (depolarized “up” states). Pyramidal-pyramidal neuron connections play a fundamental role in the generation of synchronized network events (Deuchars et al., 1994; Markram et al., 1997; Thomson and Deuchars, 1997; Morishima and Kawaguchi, 2006; Berger et al., 2010; Sippy and Yuste, 2013). Recurrent excitations occur both locally within a minicolumn and distally through cortico-cortical connections and intra-cortical horizontal fiber systems (Foehring et al., 2000; Douglas and Martin, 2004; Kalisman et al., 2005; Song et al., 2005; Perin et al., 2011). The larger number of correlated pairs and increased strength of correlations in PINK1^−^/^−^ vs. wt M1 cortex may result from (i) a difference of spontaneous activity in the network; or (ii) a change in the number and/or strength of synaptic connections (Mao et al., 2001). The former hypothesis can be ruled out because we did not identify any change of spontaneous activity (number of active cells or mean frequency of onsets). Whether the second hypothesis is valid remains to be determined and this might be a difficult task because of the very low “hit rate” for recording synaptically coupled layer V pyramidal cells in paired recordings (Markram et al., 1997). Indeed in spite of systematic recordings in various experimental conditions to facilitate the occurrence of EPSPs, we failed to evoke them by antidromic invasion of pyramidal collaterals in PINK1^−^/^−^ as in wt M1.

Several data led us to postulate that chronic L-dopa treatment of PINK1^−^/^−^ pups could reverse the excess of synchronization of the juvenile M1 network. The midbrain dopaminergic pathway is present early in development (Specht et al., 1981) and projects to superficial and deep layers of the M1 motor cortex (Descarries et al., 1987; Lewis et al., 1987). Dopaminergic receptors of the D1 and D2 subtypes are present in rodent M1 (Boyson et al., 1986; Dawson et al., 1986), dopamine decreases the probability of glutamate release in layer V pyramidal neurons via presynaptic D1 receptors (Gao et al., 2001) and dopaminergic signaling in M1 is necessary for synaptic plasticity and motor skill learning (Hosp et al., 2009; Molina-Luna et al., 2009).The lack of effect of L-dopa treatment that we found could result from an inadequate dose of injected L-dopa for an optimal spontaneous release of dopamine in the pup M1 cortical network (Stewart and Plenz, 2006, 2008) but the most likely hypothesis is that juvenile M1 hypersynchronization is independent of dopaminergic transmission. This is in agreement with our observation that the levodopa-sensitive signature of dopaminergic dysfunction in the striatum, the giant GABA_A_ currents, is not yet present in medium spiny neurons of 2 month-old PINK1^−^/^−^ mice (Dehorter and Hammond, 2014), suggesting that midbrain dopaminergic neurons do not yet dysfunction at that stage.

Desynchronization by STN-HFS resulted from the backward modulation of the M1 cortical network activity by HFS-evoked axonal spikes that antidromically propagate to a subpopulation of cortical neurons via the hyperdirect pathway (Li et al., 2007, 2012). This is in keeping with studies in hemiparkinsonian rats suggesting that HFS of the hyperdirect pathway is essential for the amelioration of PD motor symptoms (Gradinaru et al., 2009; Li et al., 2012). In our study it is unlikely that the effect of STN-HFS were mediated by orthodromic spikes in the subthalamo-cortical pathway which impinges upon layer III/IV neurons (Degos et al., 2008) because we did not record orthodromic mono or polysynaptic excitatory responses in layer V/VI pyramidal neurons. Cortico-striatal neurons whose axons do not project to the pyramidal tract (IT-type) should not be antidromically activated from the STN area and only a subpopulation of layer V/VI neurons that project to the pyramidal tract (PT-type) (Lei et al., 2004) is probably affected. The thin collaterals reaching the STN (Kita and Kita, 2012) did not always reliably transmit consecutive antidromic spikes (Chomiak and Hu, 2007; Li et al., 2012). STN-HFS-evoked antidromic spikes also propagated in some of the recurrent axon collaterals of cortico-subthalamic neurons on their way to somas of pyramidal neurons and activated synaptic transmission that impinges onto local GABAergic interneurons. These in turn decreased pyramidal neuron activity. The overall result is the desynchronization of the M1 network (Li et al., 2012; Sippy and Yuste, 2013) and the decreased influence of M1 cortical neurons on STN activity. The originality of the present result is to show that antidromic activation of a network is sufficient to reverse its abnormal pattern of synchronization and to emphasize the potential role of cortical interneurons in cortical desynchronization.

## Conflict of interest statement

Part of Constance Hammond team’s research is funded by a donation from Medtronics to Institut national de la santé et de la recherche médicale (Inserm).
